# Fine-Grained Boundary Conditions in Field-Based Routing

**DOI:** 10.3390/s24030813

**Published:** 2024-01-26

**Authors:** Jihoon Sung, Yeunwoong Kyung

**Affiliations:** 1 Department of Electrical Engineering, Korea Advanced Institute of Science and Technology, 291 Daehak-ro, Yuseong-gu, Daejeon 34141, Republic of Korea; sung.jh@kaist.ac.kr; 2 Division of Information & Communication Engineering, Kongju National University, Cheonan-daero, Cheonan 31080, Republic of Korea

**Keywords:** industrial wireless mesh network, field-based routing, boundary condition, virtual node

## Abstract

In the realm of industrial wireless mesh networks, an efficient routing protocol is highly demanded to play a crucial role in ensuring that packets are efficiently directed along shorter and congestion-free routes toward gateways. Field-based routing has emerged as a promising solution to tackle these network challenges. This routing approach draws inspiration from physics and employs a differential equation to model its behavior in finding efficient routes. Given the fundamental significance of boundary conditions in physics, where they play an essential role in shaping the solutions to the equation, exploring the impact of boundary conditions on field-based routing behavior within network domains becomes highly significant. However, despite their influence, the impact of boundary conditions has remained unexplored in existing studies on field-based routing. In this context, our work explores the boundary condition problem and introduces new advanced fine-grained boundary conditions for field-based routing. We demonstrate the superior performance of our proposed scheme, highlighting the substantial role of boundary conditions in network behavior. Our work holds significant value in that it explores the boundary condition problem, an aspect largely overlooked in previous research, and provides a viable solution, underscoring its crucial importance in routing enhancement.

## 1. Introduction

Industrial wireless mesh networks (WMNs) have gained significant attention in recent times due to their cost-effectiveness and environment adaptation capability [1,2]. According to several market reports [3,4], such industrial WMNs are also expected to have substantial growth potential as a cost-effective and practical wireless network solution for efficient communication in various industries such as manufacturing, logistics, and warehouses.

However, similar to many other emerging network technologies, industrial WMNs face challenges in areas such as routing, resource management, and security. Among these, routing is a critical issue since it plays a crucial role in ensuring the reliable and efficient delivery of data across the network [5]. As industrial WMNs continue to evolve to support a wider range of service scenarios, the development of more efficient routing protocols becomes essential to achieve better data transmission, considering the unique characteristics of these networks [6]. Various routing protocols have been introduced for these networks, such as shortest-path routing, back-pressure routing, and combination-type routing. As one of the combination-type routing protocols, field-based routing has emerged as a promising solution, flexibly balancing proximity to gateways with congestion awareness. Interestingly, field-based routing draws inspiration from physics, employing well-established differential equations such as Poisson’s or Laplace’s equations in physics [7], thus positioning itself as a physics-inspired routing protocol. The strength of this approach lies in its capacity to leverage centuries of validated scientific knowledge. In the case of field-based routing, this approach aids in deriving a novel routing metric that flexibly considers proximity to gateways and congestion when transmitting packets to gateways. The routing metric is derived by adapting the principles described by Poisson’s or Laplace’s equations to interpretations customized for network algorithms. These differential equations originally describe the interactions governing the forces of attraction and repulsion between positive and negative charges in the physical world, which has been validated over a long period. In the context of field-based routing, solving these differential equations corresponds to assigning a potential value at each node. A routing field is constructed using these potential values across all nodes. Therefore, solving these equations to determine our routing metric is essential, and it is closely intertwined with boundary conditions. Boundary conditions are restrictions applied to the equations, directly impacting the resulting solution. Well-known boundary conditions in physics include Dirichlet, Neumann, and Robin boundary conditions. In this context, it becomes evident that boundary conditions applied to boundary nodes play a crucial role within the networking domain. This is because these conditions directly shape the routing field, subsequently having a substantial influence on routing performance. Nevertheless, previous research has often underestimated the significance of boundary conditions, predominantly considering primarily simple and coarse-grained boundary conditions. This motivates us to carefully explore the boundary condition problem as a new possibility to achieve better routing performance.

Our main contribution in this paper is to propose novel fine-grained boundary conditions tailored for field-based routing, with the goal of facilitating practical field-based routing in diverse industrial environments. These fine-grained boundary conditions can be categorized into two types: outer boundary conditions applied to a subset of general mesh nodes, typically located at the outermost edges of the network, referred to as outer boundary nodes, and inner boundary conditions applied to gateways, referred to as inner boundary nodes. The proposed outer boundary conditions introduce virtual nodes for adaptability. These outer boundary conditions enable applicability to various network topologies and remain resilient even in node failure scenarios. On the other hand, the proposed inner boundary conditions assign different potential values to gateways based on the traffic load levels around them to achieve better load balancing. In summary, our research aims to enhance the performance of field-based routing by adopting the proposed fine-grained boundary conditions, which are designed to address the practical limitations of traditional field-based routing protocols.

The organization of the paper is as follows. In Section 3, we briefly review field-based routing. Then, we discuss the limitations of field-based routing in Section 4. Section 5 describes newly proposed advanced boundary conditions. In Section 6, we evaluate our proposed scheme in comparison with the existing baseline model. Finally, we conclude the paper in Section 7.

## 2. Related Work

Routing has been extensively studied as a fundamental issue in the field of networking from various perspectives, including different target network systems and metric designs. In this context, various routing protocols applicable to industrial WMNs have been also introduced in the past, each with different metrics, including shortest-path routing [8], geographic routing [9], back-pressure routing [10], and combination-type routing [11], which considers both the shortest path and congestion levels.

In contrast to traditional approaches in designing routing strategies, several field-based routing protocols have been introduced for WMNs or wireless sensor networks. These protocols draw inspiration from fundamental physical principles. For example, in [12], a mechanism targeting WMNs in underground mines considers both the distance to the gateway and the energy factor, drawing inspiration from the principle of the potential field. Additionally, anycast routing schemes for WMNs were proposed. One was designed to consider the distance and path robustness based on Fourier’s law [13], while the other was devised to consider the proximity to gateways and congestion levels through inspiration from Poisson’s Equation [14].

This approach allows these protocols to inherit the properties of their parent principles. To fully leverage the parent principle as desired, it is naturally required to understand the main logic in the laws or equations. The boundary condition problem addressed in this paper is an important problem related to the main logic, which has been unexplored so far. This motivates us to study this problem.

## 3. Revisit of Field-Based Routing Protocol

In this section, we aim to provide a brief overview of field-based routing, focusing on its fundamental concepts, operational principles, and key properties.

### 3.1. Operation Principle

Field-based routing operates by selecting the path with the steepest gradient within the routing field, which is formed based on congestion levels and proximity to gateways. Consequently, it favors the most efficient path in terms of traffic congestion levels and proximity to gateways as the next hop for forwarding packets. Initially, potential values are assigned to both inner and outer boundary nodes based on the respective boundary conditions, while initial values are assigned to the other general mesh nodes. Subsequently, each node calculates its potential value using the information received through periodic field-related information exchange, including recent traffic congestion levels and proximity to gateways, from its one-hop neighboring nodes. This process leads to the construction of the routing field. Throughout this process, the boundary conditions act as reference points for the convergence of the routing field, significantly impacting the overall routing performance.

### 3.2. Protocol Properties

The potential value of a mesh node is determined primarily by proximity to gateways and its traffic load level. The key properties of the protocol are from its simultaneous consideration of these two factors. These properties can be categorized into two main aspects:

- Proximity-aware Autonomous Load-Balancing: Field-based routing autonomously achieves load balancing because of its congestion-aware nature while considering the proximity to gateways. This facilitates traffic distribution and enables packets to effectively avoid congestion under the given proximity to gateways.
- Simplicity: The protocol achieves the aforementioned proximity-aware autonomous load-balancing by maintaining a routing field through the simple exchange of local information among only one-hop neighbors.

In the context of this paper, it is important to note that boundary conditions can significantly enhance the aforementioned advantageous characteristics, ultimately leading to improved routing performance.

## 4. Problem Statement

In this section, we primarily discuss the limitations posed by existing boundary conditions in field-based routing protocols. This discussion provides a foundation for us to elaborate on the driving factors behind the development of our proposed approach.

**Figure 1:**
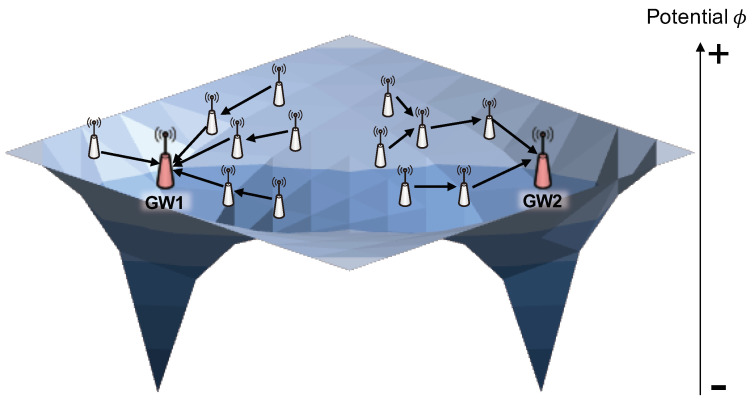
An example for operation of field-based routing.

To provide a concise overview of the existing boundary conditions, consider a scenario where a packet is sent from a mesh node with a high potential value to a gateway with the lowest potential value through a few intermediate mesh nodes, as depicted in Figure 1. As is typical in related research [12,14], the outermost nodes within the network are designated as outer boundary nodes, while mesh gateways are classified as inner boundary nodes. The potential values of all the boundary nodes are set as fixed values, with the highest potential value to outer boundary nodes and the lowest potential value assigned to inner boundary nodes. The boundary conditions exemplified by a fixed value in (1) can be expressed as follows:

(1) ϕ(B)=1, ϕ(D)=0

where ϕ(n) is a potential value at node *n*, *B* represents a set of outer boundary nodes, and *D* indicates a set of inner boundary nodes as destinations of packets. These boundary conditions, applied in previous related research, are referred to as coarse-grained boundary conditions in this paper. These coarse-grained boundary conditions correspond to Dirichlet boundary conditions [15], which assign a single fixed potential value to each point on the boundary.

The limitations of the Dirichlet-based coarse-grained boundary conditions can be described as follows. First, we explore existing outer boundary conditions. Traditionally, outer boundary nodes have been implicitly considered a subset of general mesh nodes located at the outermost edges of the given network. However, the challenge lies in defining the outermost edges, particularly in network topologies with irregular node deployments, as opposed to grid regular network topologies. To illustrate this challenge, consider a straightforward extreme example involving a grid regular network topology with a hole at its center. Here, the hole is defined as regions where no active mesh nodes are present. According to the existing implicit definition of outer boundary nodes, the mesh nodes surrounding the central hole would not be qualified as outer boundary nodes. However, it is more reasonable to categorize these mesh nodes surrounding the hole as the outer boundary nodes since their location is not fundamentally different from that of mesh nodes located at the outermost edges of the network in terms of neighbor node deployment. This highlights the need for a clear rule to determine outer boundary nodes based on neighbor node deployment rather than just relying on the physical location of the mesh nodes.

Secondly, under the existing Dirichlet-based outer boundary conditions, all outer boundary nodes are uniformly assigned the highest potential value, regardless of their surroundings. This uniformity implies that outer boundary nodes tend not to exchange packets among themselves due to their identical potential values. Consequently, this situation may lead to congestion issues because these nodes consistently forward packets into the interior of the network.

On the other hand, regarding inner boundary conditions, all gateways are assigned the same lowest potential value, regardless of the load levels in their vicinity. This approach hinders effective load balancing since it does not consider the load levels when determining the potential value. Given the importance of load balancing as a key property of routing protocols [16], it is crucial to explore new inner boundary conditions that can better support this aspect. However, when considering new inner boundary conditions, it is also essential to consider that inner boundary nodes play a crucial role as drainage points for achieving convergence in the routing field. That is, dynamically changing potential values at inner boundary nodes, which serve as drainage points for the potential field, depending on various situations can pose challenges to field convergence. Therefore, it is advisable to maintain a set of consistent potential values for stable convergence. In this context, we address inner boundary conditions as a secondary perspective in this paper, recognizing the need for a more conservative approach.

Finally, it is motivated to conduct an in-depth study to devise a solution to overcome the limitations of the existing boundary conditions, as they can directly affect routing performance.

## 5. Advanced Fine-Grained Boundary Conditions

In this section, we present a unified solution aimed at overcoming the previously mentioned limitations for existing boundary conditions, with a specific focus on the utilization of advanced boundary conditions. Prior to introducing these advanced boundary conditions, we provide a brief overview of well-known boundary conditions, such as Dirichlet, Neumann, and Robin boundary conditions, to facilitate a more systematic understanding of boundary conditions.

### 5.1. Revisit of Boundary Conditions in Physics

We start by briefly examining the definitions and unique features of well-known boundary conditions in physics [15]. Table 1 presents the mathematical expressions for these boundary conditions. Dirichlet boundary conditions specify the value of a function on boundary ∂Ω of given domain Ω in a differential equation, while Neumann boundary conditions specify the normal derivative on a boundary in the context of a differential equation. Robin boundary conditions, on the other hand, specify both the value of the function and the value of the normal derivative on the boundary simultaneously, making them a weighted combination of Dirichlet and Neumann boundary conditions.

By examining these well-known boundary conditions, we can see that Neumann and Robin boundary conditions permit changes in the potential value of outer boundary nodes based on the derivative concept, which is mathematically expressed by $\frac{\partial \phi (n)}{\partial n}$. Consequently, it becomes apparent that adopting Robin boundary conditions as new boundary conditions is the most suitable choice, as they can combine the advantages of both Dirichlet and Neumann boundary conditions.

### 5.2. Advanced Fine-Grained Boundary Conditions

#### 5.2.1. Outer Boundary Conditions

The limitations discussed in the previous section regarding outer boundary conditions can be classified into two categories: first, the absence of clear definitions for outer boundary nodes; and second, the inability to account for congestion caused by the identical fixed potential values assigned to boundary nodes. These challenges can be addressed by providing clear definitions for boundary nodes and incorporating new boundary conditions that allow for the assignment of different potential values based on congestion levels, even at the boundary nodes.

Firstly, we establish a clear definition for outer boundary nodes to categorize them consistently without any ambiguity, as described in the previous section. This definition enables each mesh node to determine its eligibility as an outer boundary node based on neighbor node deployment rather than just relying on the physical location of the mesh node. To inspect the deployment of neighbor nodes, we adopt a four-quadrant technique, a widely used method in mathematics and geometry for dividing a space around each mesh node into smaller parts. In this context, quadrants are formally defined as regional parts obtained by dividing a space into four areas by two orthogonal coordinate axes. Specifically, quadrant 1 corresponds to the top-right part, quadrant 2 to the top-left part, quadrant 3 to the bottom-left part, and quadrant 4 to the bottom-right part. It considers the challenge presented by the example of a network with a hole discussed in the previous section. Let *N* represent the set of all mesh nodes in a given network, and let $Q_{n,k}$ denote the *k*-th quadrant within the transmission range of mesh node n∈N. We assume that each quadrant includes the axis that is first encountered counterclockwise to avoid ambiguity. Additionally, amodb represents the remainder when *a* is divided by *b*, and { } indicates an empty set. With this notation, a general mesh node is qualified as an outer boundary node if one of the following four conditions is satisfied. The designation of an outer boundary node totally depends on the number of consecutive quadrants without neighboring nodes within the transmission range. This rule is formulated separately for four cases and can be expressed mathematically as shown below, where i∈{0,1,2,3}:

1. No neighbors in four consecutive quadrants:
  $Q_{n,(i\;\;\mathrm{mod}\;\;4)+1},\;Q_{n,((i+1)\;\;\mathrm{mod}\;\;4)+1},\;Q_{n,((i+2)\;\;\mathrm{mod}\;\;4)+1}$,
  and $Q_{n,((i+3)\;\;\mathrm{mod}\;\;4)+1}=\left\{\right\}$
2. No neighbors in three consecutive quadrants:
  $Q_{n,(i\;\;\mathrm{mod}\;\;4)+1},\;Q_{n,((i+1)\;\;\mathrm{mod}\;\;4)+1}$, and $Q_{n,((i+2)\;\;\mathrm{mod}\;\;4)+1}=\left\{\right\}$
3. No neighbors in two consecutive quadrants:
  $Q_{n,(i\;\;\mathrm{mod}\;\;4)+1}$ and $Q_{n,((i+1)\;\;\mathrm{mod}\;\;4)+1}=\left\{\right\}$
4. No neighbors in only one consecutive quadrant:
  $Q_{n,(i\;\;\mathrm{mod}\;\;4)+1}=\left\{\right\}$

**Figure 2:**
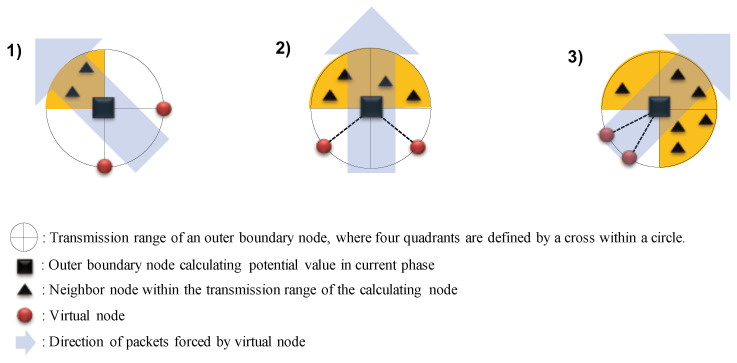
Proposed finer-grained outer boundary conditions with virtual nodes. No neighbors in (1) three; (2) two; (3) only one consecutive quadrants.

Once all mesh nodes have determined their status as either general mesh nodes or outer boundary nodes based on the rule mentioned above, the next crucial task is to implement the proposed Robin-based boundary conditions. These conditions allow for varying potential values for the outer boundary nodes. To accomplish this, we introduce the concept of virtual nodes, as depicted in Figure 2. Virtual nodes do not physically exist in the network but serve as a means to enable real outer boundary nodes to function as network boundaries, while also reflecting traffic load, similar to general mesh nodes.

We explain how to arrange virtual nodes for implementing the Robin boundary conditions and discuss their effects. All mesh nodes inspect the deployment of their neighbors and calculate potential values by placing a few virtual nodes based on the inspection results. To illustrate, we consider the scenarios shown in Figure 2. In the first case, neighbors are present only in the second quadrant. Initially, a mesh node identifies itself as an outer boundary node based on the proposed outer boundary node definition. Subsequently, it arranges virtual nodes as if they were non-existent but virtually within its transmission range. The highest fixed potential value is assigned to these virtual nodes. The outer boundary node then calculates potential values with the same considerations as general mesh nodes, leading to packet movement to the network interior direction from the outermost edges of the network, as indicated in Figure 2. The second and third cases follow the same principles as the first case, with the only distinction for the placement of virtual nodes, depending on the arrangement of neighbors. As depicted in the figure, virtual nodes are positioned along the boundary in the quadrants opposite to where actual mesh nodes are deployed. However, the conditions and methods illustrated in the figure for arranging virtual nodes along the boundary in the opposite quadrants are just one specific example, and they can be flexibly adjusted at the discretion of the network operator.

In this paper, we assume a simple virtual node deployment rule, where two virtual nodes are positioned counterclockwise along the boundary, midway between the starting and ending quadrants of the contiguous quadrants where actual mesh nodes are not deployed. In more detail, the first virtual node is positioned by adding one-third of the angular difference, counterclockwise, between the starting angle of the starting quadrant and the ending angle of the ending quadrant of contiguous quadrants where actual nodes are not deployed, starting from the starting angle of the starting quadrant. The position of the second virtual node is determined by subtracting one-third of the angular difference, counterclockwise, between the starting angle of the starting quadrant and the ending angle of the ending quadrant of the contiguous quadrants where actual nodes are not deployed, starting from the ending angle of the ending quadrant. Lastly, in cases where there are no neighboring nodes in all four quadrants, there is no viable solution, regardless of whether virtual nodes are placed or not. Therefore, in such scenarios, the node is directly defined as an outer boundary node, and the highest potential value is assigned to it. This situation is not illustrated in the figure.

The boundary conditions, involving virtual nodes, can be applied to various network topologies with irregular node deployment patterns in real-world environments, as well as simple grid-like topologies. By strategically placing virtual nodes within the transmission range of calculating mesh nodes, the proposed boundary conditions allow each mesh node to calculate potential values without any exceptions, making them highly suitable for real network environments. Note that the existing boundary conditions, which do not consider virtual nodes, could not support the calculation of potential values in all cases, especially in scenarios involving network holes. More detailed information, including the reason for this limitation of the inability to calculate potential values in certain areas, is discussed in the following section.

Fundamentally, the boundary conditions for real outer boundary nodes are equivalent to Robin boundary conditions, whereas the boundary conditions can be interpreted as the Dirichlet boundary conditions from the perspective of the virtual nodes. More precisely, the real outer boundary nodes calculate potential values by placing the virtual nodes in necessary cases as described in Figure 2, following the same principles as general mesh nodes. The boundary conditions for the real outer boundary nodes can be mathematically expressed as shown in (2).

(2) ϕ(b)=A+Bg(ϕ(v)), on b,v∈∂Ω

where ϕ(b) indicates the potential value of outer boundary node *b* while ϕ(v) represents that of virtual node *v*. *A* and *B* are simplified constant terms. Additionally, g(ϕ(v)) represents a function that indicates a change in potential value. It behaves like a derivative function with respect to ϕ(b) for given ϕ(v). This change is influenced not only by a fixed potential value by the proposed outer boundary conditions but also by the proximity to gateways. Actually, (2) is conceptually equivalent to Robin boundary conditions in Table 1.

#### 5.2.2. Inner Boundary Conditions

Inner boundary conditions are the boundary conditions applied to gateways as inner boundary nodes. As discussed in the previous section, the limitation of the existing inner boundary conditions is that they do not reflect neighboring congestion levels and assign the lowest potential value identically to all gateways. To overcome this limitation, one possible approach could involve continuously adjusting the potential values of gateways based on surrounding congestion levels, treating them similarly to general mesh nodes.

However, as mentioned in the previous section, this approach can pose challenges to field convergence since gateways play a crucial role as drainage points for the potential field. In this context, we aim to find an alternative approach as a compromise to address the limitations of existing inner boundary conditions while avoiding dynamic potential value changes for gateways during network operation.

We propose advanced Dirichlet-based boundary conditions as new inner boundary conditions. Instead of assigning the lowest potential value to all gateways, we suggest assigning different fixed potential values to each gateway. For load balancing, it is recommended to assign potential values inversely proportional to the expected congestion levels for each gateway. This rule would encourage the forwarding of a portion of packets from a congested sub-domain to another sub-domain in cases where multiple gateways are present. In this way, the proposed inner boundary conditions can lead to an improved load balancing effect while maintaining stable convergence, even with a relatively straightforward approach.

## 6. Performance Evaluation

In this paper, we consider ALFA [14] as a representative model of field-based routing protocols for performance evaluation. We briefly review the existing approach that utilizes the conventional Dirichlet boundary conditions for better understanding and subsequently evaluate a new version of ALFA, which incorporates the proposed Robin boundary conditions.

### 6.1. Review of ALFA

ALFA is a field-based routing protocol designed based on Poisson’s equation in physics, which models a distribution of electric fields across a region. This protocol design relies on a conceptual mapping between physics and network fields as follows: the medium in physics corresponds to mesh nodes in the network, (−) pole charges are analogous to mesh gateways, and (+) moving charges represent packets. According to this conceptual mapping relationship, the movement behavior of (+) moving charges towards (−) pole charges can be interpreted as the forwarding behavior of packets towards gateways. To provide a more detailed description, repulsion forces among (+) moving charges can be associated with congestion levels by packets, while attraction forces between (+) moving charges and (−) pole charges can be related to the consideration of the short path. This protocol metric shown in (3) is derived using the finite element method (FEM) [17], a widely used technique in designing distributed algorithms.

(3) $$\phi \left(x\right)=\frac{\left(\sum_{a=0}^{b-1}\frac{\left(\phi_{x,a+1}\cdot {\vec{t}}_{x,a}-\phi_{x,a}\cdot {\vec{t}}_{x,a+1}\right)\cdot \left({\vec{t}}_{x,a}-{\vec{t}}_{x,a+1}\right)}{S_{a}}+\eta q_{x}\right)}{\sum_{a=0}^{b-1}\frac{{∥{\vec{t}}_{x,a}-{\vec{t}}_{x,a+1}∥}^{2}}{S_{a}}}$$

where ϕ(x) represents the potential value of mesh node *x*, $\phi_{x,a}$ indicates the potential value of the *a*-th neighboring node from mesh node *x*, ${\vec{t}}_{x,a}$ is the relative distance vector from node *a* to mesh node *x*, and $S_{a}$ indicates the area of the *a*-th triangle element. Additionally, η is the sensitivity with respect to the number of packets in the queue of mesh node *x* and $q_{x}$ represents the number of packets in the queue of mesh node *x*.

To calculate the potential value using (3), derived from the FEM, it is necessary for each mesh node to be entirely enclosed by triangle elements as finite elements formed with their neighboring nodes within the transmission range of the node. In cases where neighboring nodes at a mesh node fail to satisfy the requirement of forming a complete triangular enclosure, virtual nodes are assigned according to the deployment rule mentioned in the previous section.

Initially, every mesh node is assigned a potential value of +1 as the highest potential value, except for gateways, which are assigned the lowest potential value within the given topology. Typically, the outer boundary nodes, located at the network boundaries, maintain a constant potential value of +1 since the Dirichlet-based outer boundary conditions assign a constant value to the boundary nodes. The potential values assigned to general mesh nodes, which are not outer boundary nodes, would vary since the values are just initial values at the initial phase.

After this initial phase, periodic hello messages are broadcasted to neighbors from each mesh node, containing its potential value and location information. Each mesh node then calculates its potential value using (3) with the received information in the hello messages. As a result, a converged potential field is constructed through several iterations, corresponding to the local equilibrium method (LEM) [18], to ensure that several iterations of (3) at each element eventually lead to a global solution.

In scenarios where congestion occurs, the shape of the potential field changes, indicating locally increased potential in specific areas. In such cases, packets would be routed away from the congested hot spot with higher potential in the next phase. This autonomous load balancing leads to efficient traffic distribution and gateway load balancing. ALFA also has the ability to adapt flexibly in the event of a node failure, promptly identifying alternative paths without incurring additional overhead. This adaptive behavior makes ALFA well suited for dynamic network environments, outperforming other routing protocols in performance in terms of packet delivery ratio and throughput.

Furthermore, ALFA combines the characteristics of both geographic routing and back-pressure routing. If η is small, ALFA behaves as a geographic routing protocol, while for large η values, it operates as a back-pressure routing protocol. In this sense, ALFA is a hybrid routing protocol capable of adapting its behavior based on network conditions.

### 6.2. Simulation Scenarios and Parameters

We utilize NS-2 [19] for performance evaluation, covering both outer and inner boundary conditions. We use two metrics for performance evaluation: overall packet delivery ratio (i.e., the ratio of the total number of successfully received packets at all the gateways to the total number of sent packets from all the sources) and aggregate throughput (i.e., the aggregated amount of traffic flowing from all the sources to all the gateways within a given period of time). The focus primarily lies on assessing outer boundary conditions, with performance evaluations for inner boundary conditions as a secondary aspect of our study. This approach considers that the varying potential values at inner boundary nodes, which act as drainage points for the potential field under various circumstances, can cause negatively impact field convergence, as discussed in Section 4.

First, to assess the performance of the proposed outer boundary conditions inspired by the Robin boundary conditions, we consider three distinct topologies: grid regular, non-grid regular, and the practical Google WiFi network [20] topologies. Additionally, we consider scenarios where network holes, which can frequently occur in real-world network environments, are present. Specifically, for the grid regular and non-grid regular topologies, we intentionally create network holes, while for the practical Google WiFi network topology, we leverage network holes that are inherently present within the topology itself, without artificially creating new ones. It is worth noting that conducting performance evaluations for networks with holes allows us to derive results that are more representative of real-world conditions.

Regarding potential values, we assign 0 as the lowest potential value to gateways, following the convention in conventional ALFA. This is because we aim to purely assess the performance improvement by the proposed outer boundary conditions. In the existing ALFA using the Dirichlet boundary conditions, a +1 potential value is assigned only to outer boundary nodes within the outermost area, indicating their highest potential value. Additionally, the highest potential value is allocated to mesh nodes where potential value calculation is not feasible due to the absence of full enclosure by triangles formed with their neighboring nodes. However, in the new version of ALFA using the proposed Robin boundary conditions, a +1 potential value is assigned to the generated virtual nodes.

The new version of ALFA, with the proposed outer boundary conditions, is then compared to the existing ALFA, which utilizes the conventional Dirichlet boundary conditions traditionally used in existing field-based routing in terms of packet delivery ratio and throughput.

Second, we conduct performance evaluations of the proposed inner boundary conditions. We use the non-grid regular topology as a representative case to highlight the impact on performance evaluation. The new version of ALFA, incorporating the proposed inner boundary conditions that allow each inner boundary node to have a different potential value, is compared to the existing ALFA, which utilizes the conventional boundary conditions traditionally used in existing field-based routing. In existing field-based routing, the same potential value is assigned to all the gateways acting as inner boundary nodes.

As our simulation parameters, we set the maximum queue size to 100, and η is set to $1\times 10^{-4}$, which is empirically obtained when the best performance is observed through various simulations. All data packets are UDP packets with 1000 bytes, and the packet generation follows a Poisson distribution with a rate under the channel bandwidth of 2 Mbps. While it is possible to set arbitrary numbers directly as the packet generation rate without any specific qualitative meaning, we introduce an offered load expressed as a percentage to determine the rate in the Poisson distribution. Specifically, in an ideal scenario without contention, we simply consider the maximum network-wide allowable load as 100%, calculated as the product of the channel bandwidth and the number of gateways. We then adjust the Poisson distribution rate to align with the specified offered load, which results from UDP packet generation by all source nodes. This calculated rate is then applied to the simulation. However, it is up to the user’s discretion to choose how to set the rate. The transmission range and interference range are set to 250 m and 550 m, respectively. We also enable RTS/CTS and hello-message-jittering. Default values are applied to any other parameters or models not explicitly mentioned. To analyze the effects of our proposal, we measure the packet delivery ratio and throughput over a 600-s period in the middle of a 1000-s simulation. To ensure the reliability of the results, all simulations are repeated 10 times per scenario, and the averages are used in the graphs, with the standard deviation represented as vertical bars.

### 6.3. Simulation Results

#### 6.3.1. Outer Boundary Conditions

**Figure 3:**
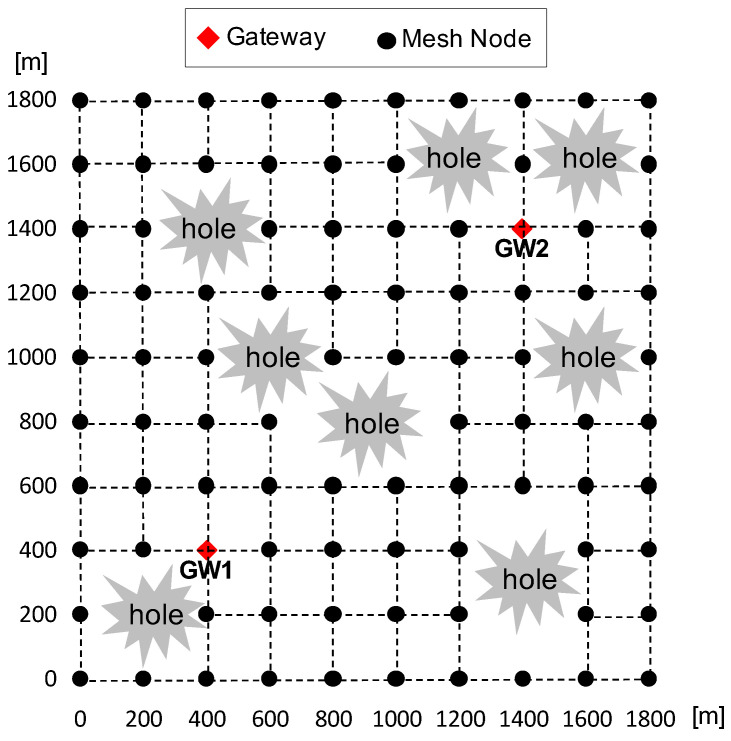
Grid regular topology with holes.

We begin by examining the results for the grid regular network topology with holes, which represents the simplest scenario, as shown in Figure 3. Note that the areas labeled as hole in the figure are just one example. In actual simulations, these holes were randomly selected. Simulations are conducted with a 30% offered traffic load generated by randomly selecting 10 mesh nodes.

**Figure 4:**
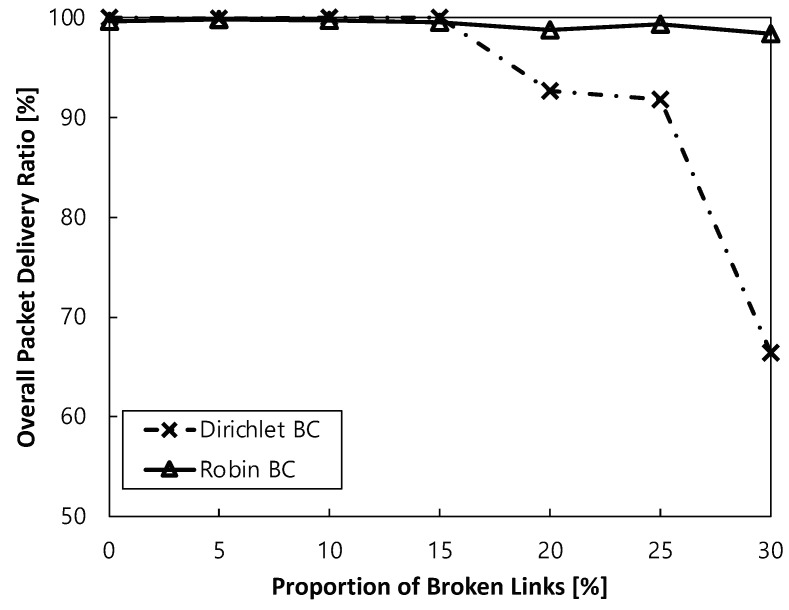
Overall packet delivery ratio under different outer boundary conditions with respect to the ratio of broken links.

Figure 4 shows the overall packet delivery ratio under different outer boundary conditions with respect to the broken link ratio. Here, the broken links are defined as the links which are not normally connected to neighbors due to the holes. Note that our performance evaluation is based on the broken link ratio rather than the number of failed nodes. This approach allows us to fairly consider various scenarios, including simultaneous failures of neighboring nodes and distant nodes. When neighboring nodes fail simultaneously, there are relatively fewer broken links, resulting in minimal disruption to packet transmission. However, when distant nodes fail simultaneously, more broken links may occur, presenting more significant challenges in packet delivery. Considering these factors, measuring performance in terms of the broken link ratio can provide a more fair assessment in the evaluation. When considering the simplest scenario of the grid regular topology, we focus solely on the overall packet delivery ratio results, without including additional metrics such as throughput. This approach allows us to provide a clear and concise illustration of the impact of network holes as the first demonstration of our concept.

Finally, the performance of the proposed ALFA by Robin boundary conditions (referred to as Robin BC) is significantly better than the conventional ALFA with Dirichlet-based boundary conditions (referred to as Dirichlet BC) as the ratio of broken links increases, with a maximum difference of 48% in terms of the overall packet delivery ratio. Specifically, up to a broken link ratio of 15%, there is little difference in performance between the two schemes. However, the performance gap begins to widen: at 20%, it is 7%; at 25%, it is 8%; and at 30%, it increases to 48%. The reason is that a routing field is not normally converged due to the absence of a sophisticated method to calculate potential values at the vacant regions by the existing simple Dirichlet-based boundary conditions to assign a fixed potential value to boundary nodes. On the other hand, there is no such limitation imposed by the proposed boundary conditions, as all the boundary nodes can better adapt to various situations by establishing a stable routing field in any scenario. In other words, ALFA with the proposed boundary conditions is more robust for the networks with holes.

**Figure 5:**
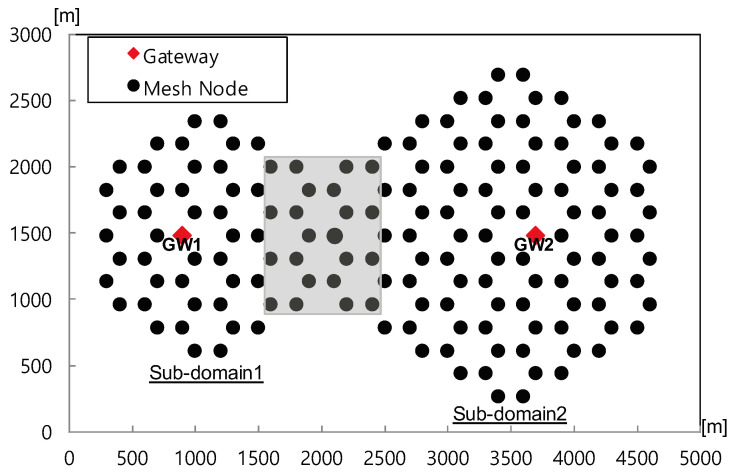
Non-grid regular topology with holes.

Next, we conduct simulations in a non-grid regular network topology with several holes to evaluate the proposed outer boundary conditions, similar to the grid scenario. In detail, we consider a non-grid regular network consisting of 154 mesh nodes and two gateways distributed across two sub-domains, with each sub-domain having a gateway deployed, as shown in Figure 5. These sub-domains consist of one small domain (referred to as sub-domain 1) and one large domain (referred to as sub-domain 2). The holes within the network are randomly generated with a broken link ratio of 30% per simulation run. We randomly select 10 mesh nodes at the intersections (highlighted in grey) of the sub-domains to create a congested hot spot by generating highly loaded traffic with an 80% offered load. Additionally, background traffic with a 10% offered load is generated by randomly selected 20 nodes, excluding the 10 mesh nodes chosen for generating highly loaded traffic at the intersections of sub-domains.

**Figure 6:**
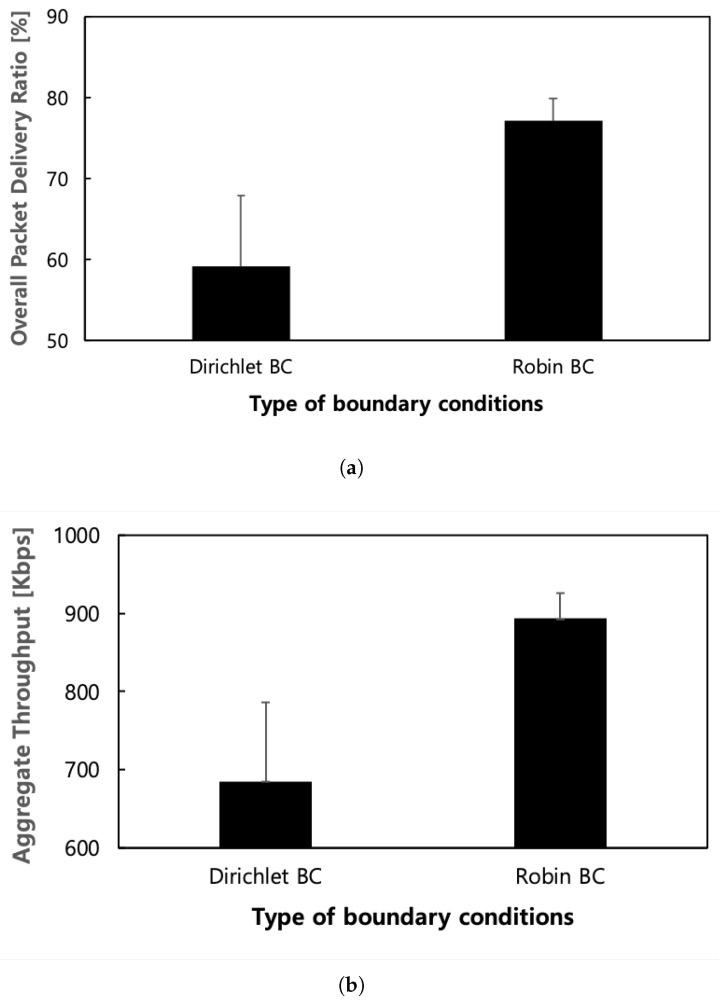
Performance according to kind of outer boundary conditions in non-grid regular topology with holes. (**a**) Overall packet delivery ratio; (**b**) Aggregate throughput.

As shown in Figure 6, the protocol with fine-grained Robin boundary conditions exhibits superior performance to the protocol with coarse-grained Dirichlet boundary conditions, achieving an aggregate throughput exceeding 200 Kbps and increasing the packet delivery ratio by 17% on average. Additionally, the proposed scheme, corresponding to the former, demonstrates approximately three times better performance in standard deviation compared to the latter, corresponding to the conventional scheme in terms of both the overall packet delivery and aggregate throughput. This indicates that the protocol with coarse-grained Dirichlet boundary conditions performs poorly in networks with holes because the holes obstruct the diffusion of potential. As a result, the routing field is constructed unstably. In contrast, the protocol using fine-grained boundary conditions exhibits particular strength in networks that include holes as it can stably achieve potential field convergence in any scenario.

**Figure 7:**
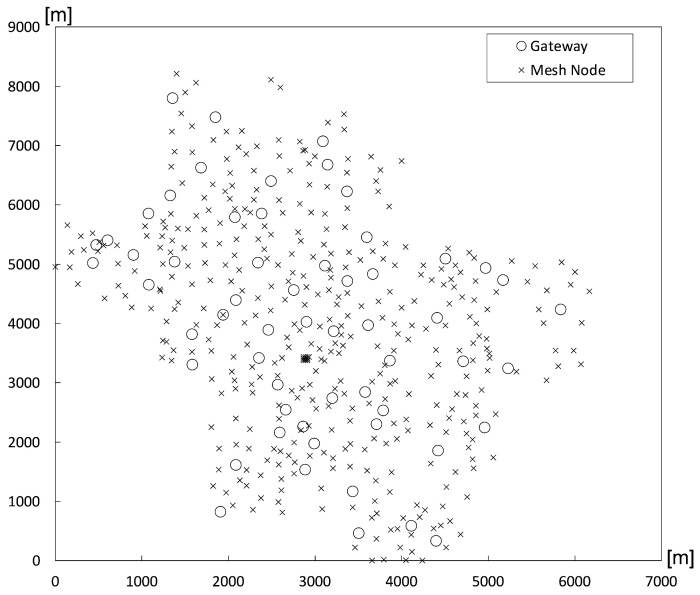
Google WiFi network topology.

**Figure 8:**
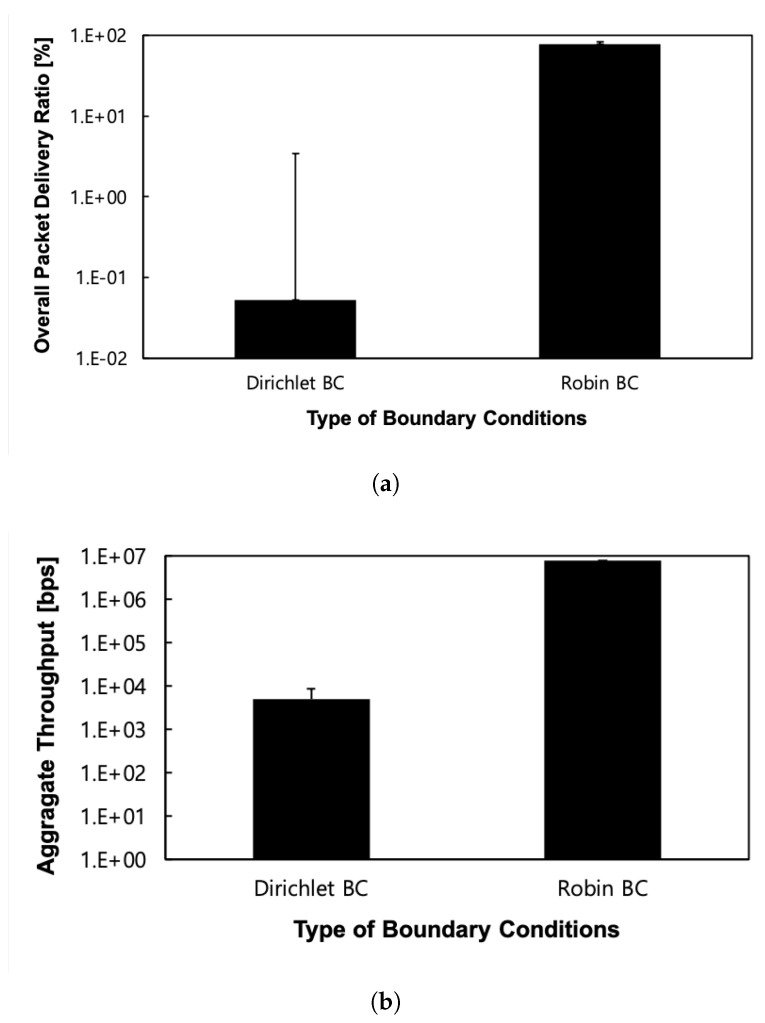
Performance according to the kind of outer boundary conditions in the Google WiFi network topology. (**a**) Overall packet delivery ratio; (**b**) Aggregate throughput.

Lastly, we evaluate our proposed scheme in a practical Google WiFi network topology [20] with irregular node positions, as shown in Figure 7. This real-world environment naturally contains network holes, making the convergence of a routing field challenging. The protocol with the proposed Robin boundary conditions consistently outperforms the protocol with the existing Dirichlet-based boundary conditions in terms of the overall packet delivery ratio and aggregate throughput, as shown in Figure 8. Specifically, significant improvements are observed over 1000 times in terms of both the overall packet delivery ratio and aggregate throughput on average, with standard deviations showing 16% and 35% smaller results in the same metrics, respectively. The existing Dirichlet-based boundary conditions fail to construct a normal routing field because they cannot form the necessary triangles required for potential value calculation. This limitation of the existing boundary conditions is effectively addressed by the proposed Robin boundary conditions.

Notably, a performance gap between the proposed Robin boundary conditions and the existing Dirichlet boundary conditions is substantial in both packet delivery ratio and throughput, as evident from the exponential scale on the y-axis. This highlights the significant weakness of the existing Dirichlet boundary conditions in real-world scenarios with irregular node deployments and multiple naturally occurring holes, while the proposed Robin boundary conditions demonstrate robustness in such contexts.

#### 6.3.2. Inner Boundary Conditions

As previously mentioned, our evaluation of inner boundary conditions focuses exclusively on the non-grid regular topology as a representative case that best demonstrates their impact. To purely assess the effects of the proposed inner boundary conditions, we eliminate other factors in our simulations. Specifically, we assume a network topology with no broken links and apply the existing Dirichlet-based outer boundary conditions. In this configuration, we assign a 20% offered load to sub-domain 1 and an 80% offered load to sub-domain 2 by randomly selected nodes, thereby creating distinct loads for each sub-domain. Additionally, we set the potential value of the gateway in the sub-domain 1 to 0, while in the sub-domain 2, it is set to 0.5. This adjustment considers the difference in the number of nodes between the two sub-domains, ensuring that the expected load difference also is twice. By doing so, we aim to demonstrate the performance difference between ALFA models using conventional coarse-grained Dirichlet-based and advanced fine-grained Dirichlet-based inner boundary conditions.

**Figure 9:**
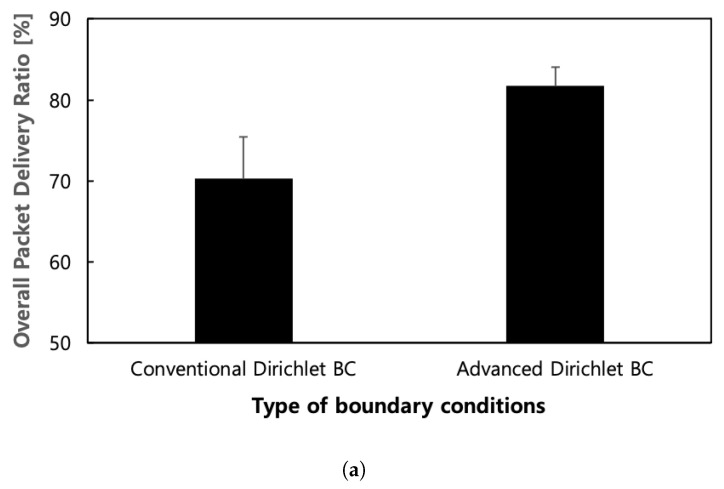

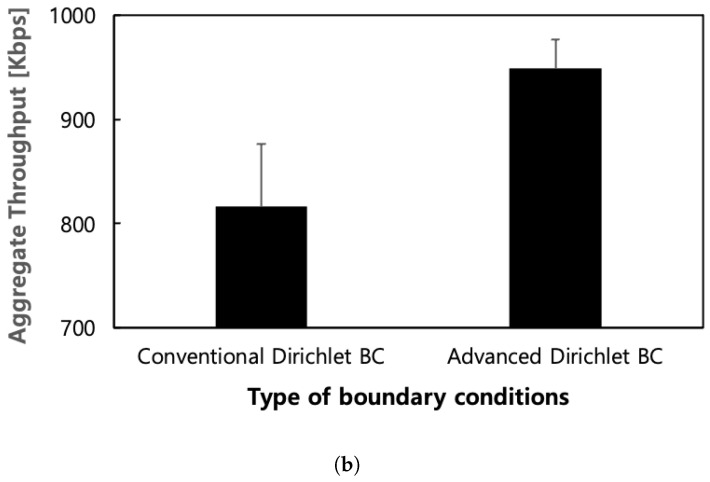
Performance according to the kind of inner boundary conditions in the non-grid regular topology. (**a**) Overall packet delivery ratio; (**b**) Aggregate throughput.

Our findings, depicted in Figure 9, reveal that the new version of ALFA with advanced fine-grained inner boundary conditions outperforms the existing ALFA with the coarse-grained inner boundary conditions in terms of the overall packet delivery ratio and aggregate throughput. We observe an approximate increase of 130 Kbps in aggregate throughput and a 10% boost in overall packet delivery ratio, on average. Additionally, approximately two times better results are observed for both metrics in terms of standard deviation. This demonstrates that the advanced inner boundary conditions enable ALFA to better adapt to the surrounding traffic load levels. Fundamentally, the routing field directs a portion of the traffic from sub-domain 2 towards sub-domain 1. It is worth noting that the observed improvement, while not dramatic, is indeed significant, highlighting the impact of modifying the inner boundary conditions within the same protocol.

## 7. Conclusions and Future Work

In this paper, we propose new fine-grained boundary conditions for field-based routing protocols as a promising enabler to enhance their applicability in both present and future industrial WMNs. These proposed boundary conditions include novel outer boundary conditions inspired by Robin boundary conditions in physics and inner boundary conditions reinterpreted from Dirichlet boundary conditions in physics. Additionally, we introduce the concept of virtual nodes to implement these new fine-grained boundary conditions. Ultimately, our proposed boundary conditions significantly improve routing performance. Rigorous simulations conducted on various network topologies, including practical examples like Google WiFi mesh networks, as well as grid regular network topologies, demonstrate that field-based routing with these fine-grained boundary conditions outperforms existing protocols using coarse-grained boundary conditions. Regarding the outer boundary conditions, in the grid regular network topology scenario, a maximum performance difference of 48% in the overall packet delivery ratio was observed. In the non-grid regular topology scenario, gains of 200 Kbps and 17% were observed in overall packet delivery ratio and aggregate throughput, respectively. In the case of Google WiFi network topology, significant improvements of over 1000 times in terms of the same metrics were observed. On the other hand, regarding the inner boundary conditions, an approximate increase of 10% in overall packet delivery ratio and 130 Kbps in aggregate throughput was achieved.

However, it is important to recognize the limitations of our study. One of the key limitations is the absence of an explicit method to determine the optimal value of η, which represents sensitivity to the number of packets in the queue of mesh nodes. Currently, this parameter tuning heavily relies on empirical results obtained through extensive simulations. Moreover, it is noteworthy that dealing with this challenge is not unique to our study, but is a common issue in algorithms with tuning parameters.

Moving forward, in the context of future research directions, we recognize the imperative need to explore alternative methods for determining the optimal value of η without solely relying on empirical results obtained through extensive simulations. One possible avenue of exploration involves investigating heuristic configurations that assign varying weights based on network traffic load or queue lengths. This approach, although it may seem immature, may lead to more advanced research efforts aimed at discovering superior methodologies for parameter optimization.

## Figures and Tables

**Table 1:** Definition of well-known boundary conditions.

Well-Known Boundary Conditions	Definition of Mathematical Expression
Dirichlet Boundary Conditions	ϕ(n)=f(n),∀n∈∂Ω
Neumann Boundary Conditions	$\frac{\partial \phi (n)}{\partial n}=f(n),\forall n\in \partial \Omega$
Robin Boundary Conditions	$\alpha \phi \left(n\right)+\beta \frac{\partial \phi (n)}{\partial n}=f\left(n\right),\forall n\in \partial \Omega$

Ω: given domain/∂Ω: boundary in given domain/*n*: node/α,β,f: functions (regarded as constant functions in this paper for simplicity unless otherwise specified).

## Data Availability

Data are contained within the article.
